# Clinical and genetic findings in Hungarian patients with X-linked juvenile retinoschisis

**Published:** 2008-12-12

**Authors:** B. Lesch, V. Szabó, M. Kánya, G.M. Somfai, R. Vámos, B. Varsányi, Zs. Pámer, K. Knézy, Gy. Salacz, M. Janáky, M. Ferencz, J. Hargitai, A. Papp, Á. Farkas

**Affiliations:** 1 Department of Ophthalmology, Semmelweis University, Budapest, Hungary; 2 Department of Ophthalmology, Medical Faculty of the University of Pécs, Pécs, Hungary; 3 1^st^ Department of Pathology and Experimental Cancer Research, Semmelweis University, Budapest, Hungary; 4 OBIK Medical Biotechnology Innovation Center Inc., Budapest, Hungary; 5 Department of Ophthalmology, Medical Faculty of the University of Szeged, Szeged, Hungary

## Abstract

**Purpose:**

To determine clinical phenotypes, examine the age dependency of X-linked juvenile retinoschisis (XLRS), and identify mutations in the retinoschisis1 gene (*RS1*) in 13 Hungarian (Caucasian) families with this disease.

**Methods:**

This study included 72 members in 13 families. Complete ophthalmological examinations, including optical coherence tomography (OCT) and full-field and multifocal electroretinography (ERG), were performed on 20 affected males, 13 female carriers, and 27 healthy controls. The patients were divided into two age groups (Group I <25 years and Group II >25 years), retrospectively, to assess the possible effects of age. Correlations among genotype, age, best corrected visual acuity (BCVA), OCT, and ERG results were analyzed. A modified classification scheme was done to identify the different phenotypes of the disease. In each of the 72 family members and 100 age-matched male controls, all exons and introns of *RS1* were amplified by polymerase chain reaction (PCR) and directly sequenced.

**Results:**

Foveal retinoschisis was detected in 25 eyes (62.5%) of patients by funduscopy, and in 29 eyes (72.5%) by OCT, while macular lamellar schisis was recognizable only by OCT in 30 eyes (75%) of patients. Foveal thickness (FT) and total macular volume were significantly increased in younger (Group I) patients only. For patients younger than 26 years, large inner nuclear central cysts were observable by OCT, while after 26 years, foveas were atrophic. White flecks and dots, which were like that seen in fundus albipunctatus, were detected in both eyes of one patient. In both patient groups, characteristically decreased b-waves of standard combined ERG were recorded without any significant difference between the patient groups. The BCVA and ERG parameters of all patients and the OCT of younger patients were significantly worse (p<0.05) than those of age-matched controls. A significant difference between the two age groups was found in case FT, total macular volume, and amplitudes of rod b-wave only. Moderate negative correlation (r=-0.54, p<0.001) was detected between age and FT, while only low negative correlation (r=-0.33, p<0.05) was detected between age and standard combined b-wave amplitudes of full-field ERG. BCVA LogMAR did not show any obvious correlation with age (r=-0.14, p=0.39) or with the type of mutation. Nine different mutations were identified in 25 male patients and 31 female carriers of 13 families: six known and one novel missense mutation (c.575C>T, p.Pro192Leu), one insertion mutation (c.579dupC, p.Ile194Hisfs29ext43), and one frameshift, causing splice site mutation (c.78+1G>C) were detected. These mutations were absent in the 100 age-matched male control samples.

**Conclusions:**

Foveal cystic schisis was found more often by OCT than by funduscopy (+10%), while flat macular lamellar schisis was recognizable only by OCT. Advancing age inversely influenced the size of cavities (FT), and standard combined b-wave amplitudes of full-field ERG, while BCVA, response density, and implicit times of multifocal electroretinography did not show any obvious correlation with age. The atrophic stage of the disease was observable after 26 years of age. The lesions that appeared to be indicative of fundus albipunctatus were proven to be palisades between the splitted retinal layers. Our modified classification scheme was helpful in assessing the prevalence of disease types. In these Hungarian patients, one novel and eight known mutations were detected. The distribution of mutations in *RS1* was different to that reported in the literature, because the greatest number of different mutations was in exon 6 instead of exon 4. Two mutation hot spots were found: between c.418–422 in exon 5 and between c.574–579 in exon 6. Genotype-phenotype correlation was not demonstrable.

## Introduction

**Figure 1:**
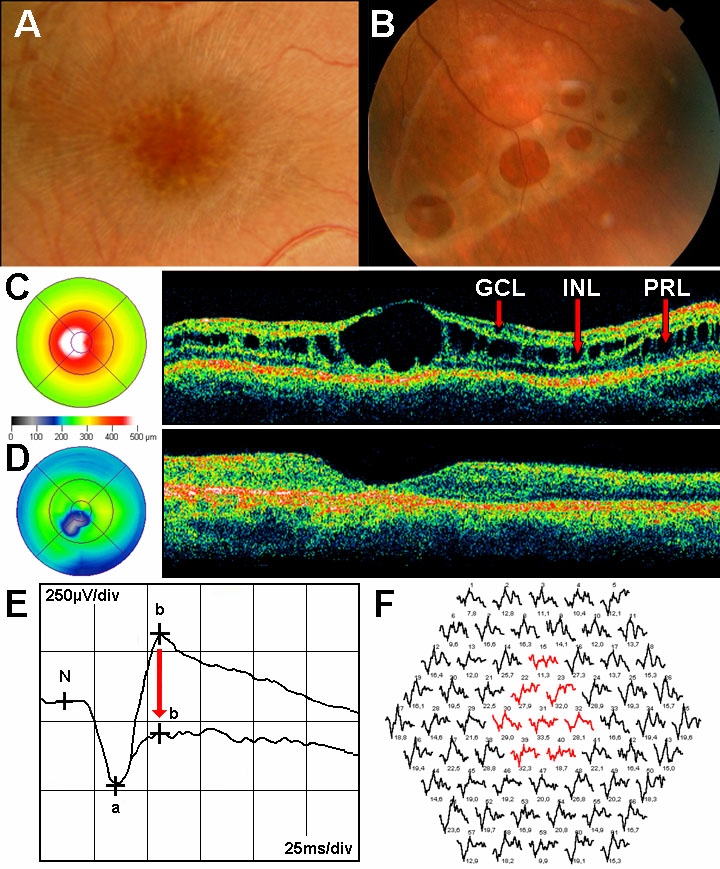
Characteristic signs of XLRS. **A:** Fundus photograph of patient IV:2 of family 1 showing foveal schisis with spoke-wheel pattern. **B:** Fundus photograph of left eye of patient III:3 of family 3 showing bullous infero-temporal retinoschisis. **C:** OCT image (scan length: 6 mm, horizontal section) of patient IV:2 of family 1 showing foveal cystic retinoschisis in three different retinal layers (marked by red arrows) and the color-coded foveal thickness maps showing the eccentric fixation. GCL indicates ganglion cell layer, INL represents inner nuclear layer and PRL refers photoreceptor layer. **D:** OCT image of patient III:2 of family 5 showing foveal atrophy and the color-coded foveal thickness map showing the eccentric fixation. **E:** Standard combined response of full-field ERG showing decreased b-wave amplitude (“negative type” ERG) related to normal (see red arrow). **F:** Multifocal ERG with 61 first-order kernels showing decreased b-(P1) wave amplitudes in the central rings (marked by red).

X-linked juvenile retinoschisis (XLRS; OMIM: 312700) is one of the most common X-linked recessively inherited, bilateral, progressive vitreoretinal dystrophies. Limited almost exclusively to males, the incidence is between 1:5,000 and 1:25,000 [1–3]. Macular changes are present in almost all cases [4]. In the fundi, radially oriented intraretinal foveomacular cysts are seen in a spoke-wheel configuration, with the absence of foveal reflex in most cases (Figure 1A). In addition, approximately half of cases have bilateral peripheral retinoschisis in the inferotemporal part of the retina (Figure 1B) [4]. Aside from the typical fundus appearance, strabismus, nystagmus, axial hyperopia, defective color vision (red-green dyschromatopsia) and foveal ectopy can be present [4,5]. The most important complications were vitreous hemorrhage, retinal detachment, and neovascular glaucoma [1,4,6]. The diagnosis of the disease is most often made in boys aged 5–10 years, who may also exhibit symptoms of uncorrectable visual disturbance and reading difficulties [4].

Optical coherence tomography (OCT) images can be variable depending on the disease stage. The cysts are located in different layers of the retina (Figure 1C), namely the nerve fiber (NFL), ganglion cell (GCL), inner nuclear (INL), outer plexiform (OPL), outer nuclear (ONL) and photoreceptor layers (PRL) [7–9]. In the course of time the coalescing cysts form a large central cavity (Figure 1C), which can pass into a nonspecific macular atrophy (Figure 1D) [3,5,10,11]. Disease severity and progression are highly variable.

Full-field electroretinograms (ERGs) of the affected individuals are characterized by predominant reduction of standard combined b-wave amplitude (negative-type ERG: b-wave to a-wave ratio less than or equal to 1) in the dark-adapted eye (Figure 1E) [2,12]. The a-wave may also be reduced with disease progression due to the increasing retinal pigment epithelium (RPE) atrophy [4,13]. The response densities of multifocal ERG (mfERG) diminish mainly in the central rings (Figure 1F) and the implicit times are extended. Thus, ERG is a useful diagnostic method in almost all cases [2].

**Figure 2:**
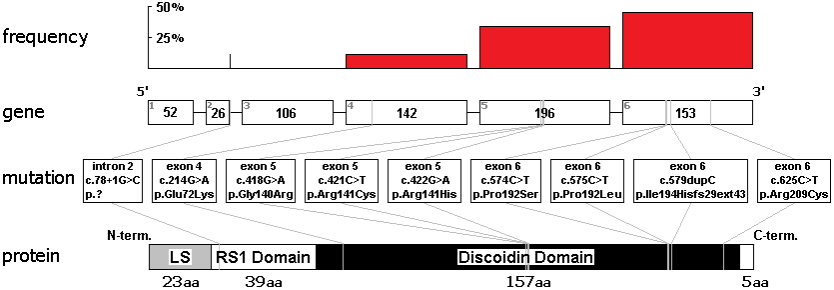
Identified mutations, their frequency in the *RS1* gene, and the retinoschisis protein. In the gene diagram, exons are represented by bars (gray numbers indicate their numbers; black numbers indicate their size in nucleotides), and introns by lines. Mutations and their localizations in the gene and the protein are marked by gray lines. In the protein diagram, leader sequence is abbreviated LS. Frequency of different types of mutations within each exon and intron are indicated by red bars.

XLRS is caused by mutation in the *RS1* gene (localization: Xp22.2-p22.1, GenBank AF014459), which was identified by positional cloning in 1997 (Figure 2) [14]. This is the only known gene to be associated with XLRS so far [15]. *RS1* has six exons and encodes a 224 amino acid (AA) secretable extracellular adhesion protein, called retinoschisin (RS1), which is primarily present in photoreceptors and in bipolar cells [6]. It may be involved in cellular adhesion and cell-cell interactions on membrane surfaces [16]. Retinoschisin includes a secretory leader sequence (LS; 23 AA), an Rs1 domain (Rs1D; 39 AA), a highly conserved discoidin domain (a main structural feature of RS; 157 AA), and a C-terminal segment (5 AA) [2,16–19]. The discoidin domain is essential for the normal development of the retina, and if functional RS1 is lacking, schisis develops in different retinal layers [14,15].

## Methods

Informed consent was obtained from all participants after a full explanation of the nature and possible consequences of the study. All studies were conducted in accordance with the tenets of the Declaration of Helsinki. Molecular genetic examinations were approved by the Ethics Committee for Human Genome Research of Semmelweis University.

### Patient and control groups

**Figure 3:**
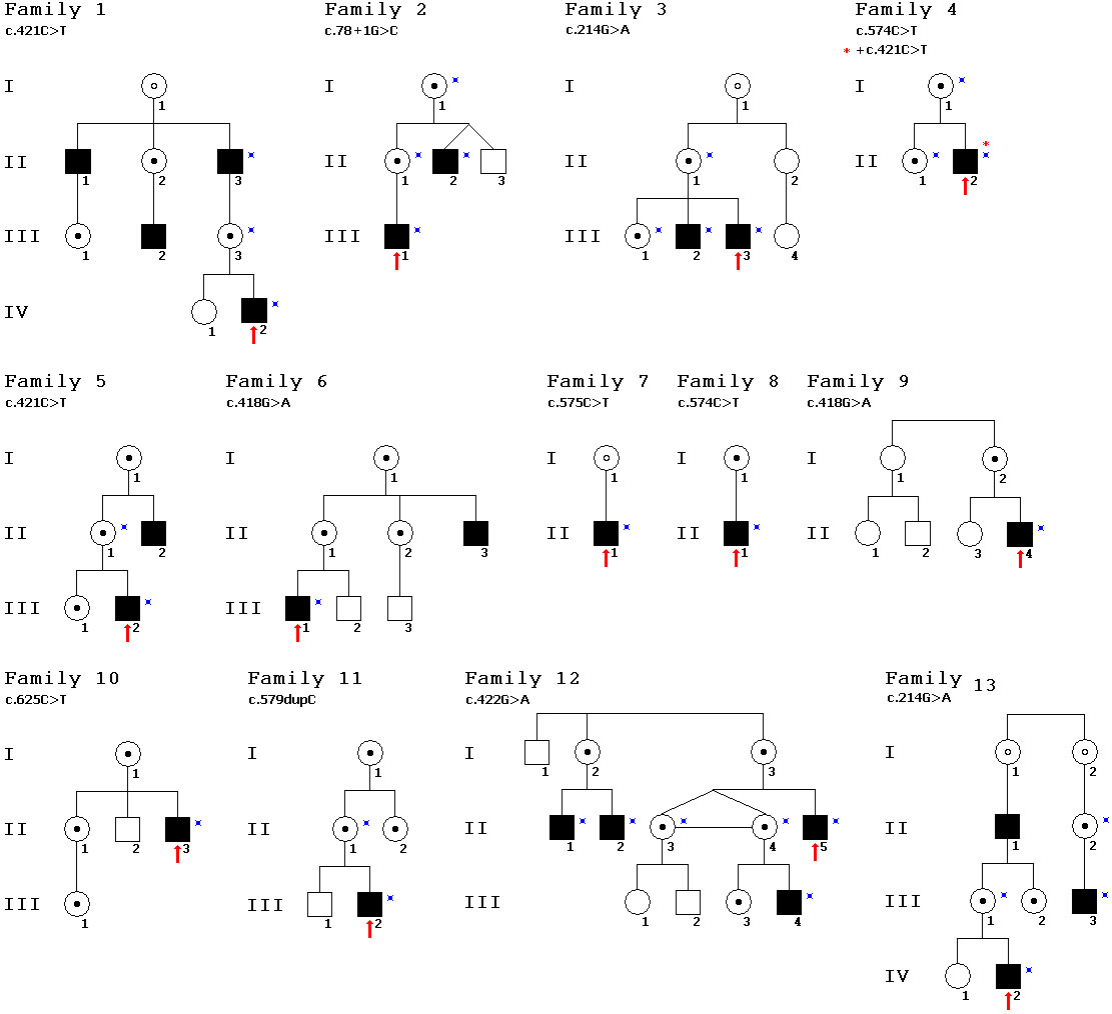
Pedigrees of 13 Hungarian families with XLRS and identified mutations in the RS1 gene. Black boxes represent affected males, while circles with a black dot in the center represent carrier females by sequence analysis. Circles with an open dot represent females those who are expected to be obligate carrier females. Red arrows point to probands. Slashed boxes are deceased family members. Blue stars mark family members who underwent complete clinical examinations. The mutation screening of RS1 was performed in all family members except probable XLRS carriers, who did not want or could not take part in the study. Patients II:2 and II:3 in family 2 are fraternal twins, while patients II:3 and II:4 in family 12 are identical twins.

**Figure 4:**
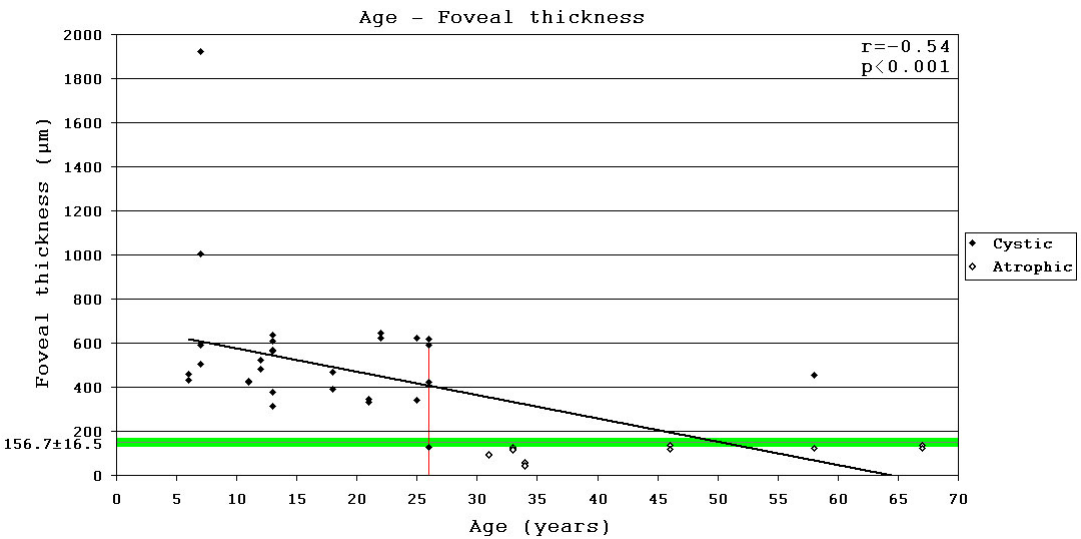
Correlation between age and foveal thickness in patients with XLRS. Black dots indicate eyes in the cystic stage, white dots represent eyes in the atrophic stage. Green-highlighted horizontal line represents the normal foveal thickness±SD. Red vertical line represents the time (26 years of age) after which no cysts were observed. Moderate negative correlation was detected between age and foveal thickness.

In all, 72 members (mean age ±SD: 32.6±19.1 years) in 13 Hungarian families were involved in the present study (Figure 3). Of the 72 family members, 20 male patients (mean age ±SD: 24.5±16.8 years) and 13 asymptomatic female carriers (mean age±SD: 37.7±13.3 years) took part in all clinical examinations: visual acuity tests, indirect ophthalmoscopy, slit lamp biomicroscopy, full-field and mfERG, and OCT. The patients were divided into two groups (Group I aged <25 years and Group II aged >25 years), retrospectively, on the basis of our observation that the cystic form of the disease manifest before 26 years of age, while the atrophic form occurs after 26 years (Appendix 1, Figure 4). In this way we could examine the possible functional effects of morphological changes and the effects of age. All 72 family members participated in molecular genetic examinations.

A control group of 27 healthy age-matched normal subjects (mean age ±SD: 20.1±8.2 years) was examined using the same clinical protocol. To confirm the pathogenetic effects of the identified mutations a control group of 100 healthy age-matched normal male subjects (mean age±SD: 22.4±9.3 years) was examined using the same molecular genetic protocol. BCVA logarithm of the Minimum Angle of Resolution=logarithm of the reciprocal of Snellen’s fraction (LogMAR units) was 0.0 in all controls, and no visual disturbances were found.

### Clinical examinations

All study participants underwent standard ophthalmological examinations. BCVA was determined using the Snellen chart then converted to LogMAR units. Dilated indirect ophthalmoscopy of the posterior pole and the periphery was performed with fundus photography.

### Optical coherence tomography

OCT was performed in both eyes of the family members using six 6 mm long OCT scans centered on the fovea by a Stratus OCT device (Carl Zeiss Medical Inc., Dublin, CA). The foveal thickness (FT) was measured manually in the center of the fovea with the more accurate caliper technique, while total macular volume (TMV) was calculated automatically by the built-in software of the device. To determine eccentric fixation, we displayed the FT on a color-coded map; the center of the map showed the fixation point (Figure 1C-D). To assess the prevalence of the different phenotypes of the disease, we modified and extended the Prenner “Classification scheme for XLRS” (Table 1) [20].

**Table 1:** Classification scheme of XLRS

**XLRS form**	**Type**	**Foveal cystic schisis (clinical and OCT exam)**	**Macular lamellar schisis (OCT exam)**	**Peripheral schisis (clinical exam)**	**BCVA LogMAR (mean±SD)**	**Prevalence (percent)**
Cystic	Type 1 Foveal	+	-	-	0.22	2.5
	Type 2 Lamellar	-	+	-	0.25±0.07	5
	Type 3 Foveolamellar	+	+	-	0.38±0.13	57.5
	Type 4 Complex	+	+	+	0.47±0.17	12.5
	Type 5 Foveoperipheral	+	-	+	-	-
	Type 6 Peripheral	-	-	+	0.85±0.21	5
Atrophic	Type 7 Nonspecific	-	-	-	0.28±0.26	17.5

The table shows the modified classification scheme of XLRS to assess the different phenotypes [20]. The scheme of classification was devised that incorporated both clinical and OCT findings. There are 6 cystic subtypes and the atrophic type. Type 3 was the most common phenotype of the disease. The prevalence of phenotypes is based on 40 eyes from 20 patients having XLRS. The 2.5 value of prevalence is equal to 1 eye. SD represents standard deviations.

### Electroretinography

For each subject, ERG was performed (RetiPort ERG system, Roland Consult, Wiesbaden, Germany) using corneal “jet” contact lens electrodes, according to International Society for Clinical Electrophysiology of Vision (ISCEV) standards. Amplitudes and implicit times of scotopic and photopic full-field ERG responses were measured, and the ratio of standard combined b- and a-wave amplitudes was calculated. During mfERG the central 60° diameter part of the retina was stimulated monocularly by 61 hexagons shown on a 21” CRT display, under photopic conditions. Amplitudes and implicit times of the b-waves (P1) of first order kernels (FOK) were measured. For each patient, response densities (RD; nV/deg^2^) in the five concentric rings were calculated. All the amplitudes and implicit times were compared with the mean values±SD of the normal age-matched controls.

### Genetic examination

Genomic DNA was extracted from peripheral blood leucocytes using the QIAamp DNA Mini Kit (Qiagen GmbH, Hilden, Germany). PCR was performed using standard protocols in a Thermo Hybaid PxE thermal cycler (Thermo Hybaid, Franklin, MA) with oligonucleotide primers published previously by the RS Consortium [15]. The amplicons were analyzed on 1% agarose gels and stained with ethidium bromide and purified using Roche High Pure PCR Purification Kit (Roche Diagnostics GmbH, Mannheim, Germany) according to the manufacturer’s protocol. Exons and the flanking intronic regions of *RS1* was sequenced by direct nucleotide sequencing using the Big Dye Terminator Cycle-Sequencing v3.1 Kit (Applied Biosystems, Foster City, CA) and run on an automated sequencer (ABI PRISM® 310 Genetic Analyzer, Perkin Elmer™; Applied Biosystems). The results were compared with the reference sequence of the X-linked retinoschisis sequence variation database (dmd).

### Statistical analysis

To assess significant changes between patients and control persons, we performed ANOVA (one-way ANOVA) followed by Newman-Keuls post hoc analysis. Significance was accepted at the p<0.05 level. Correlations (Spearman rank) were calculated between age, BCVA, OCT, and ERG parameters. For statistical analysis the Statistica 6.0 (Statsoft, Inc., Tulsa, OK) and Excel (Microsoft^®^ Corp.) software packages were used.

## Results

### Clinical examination

**Figure 5:**
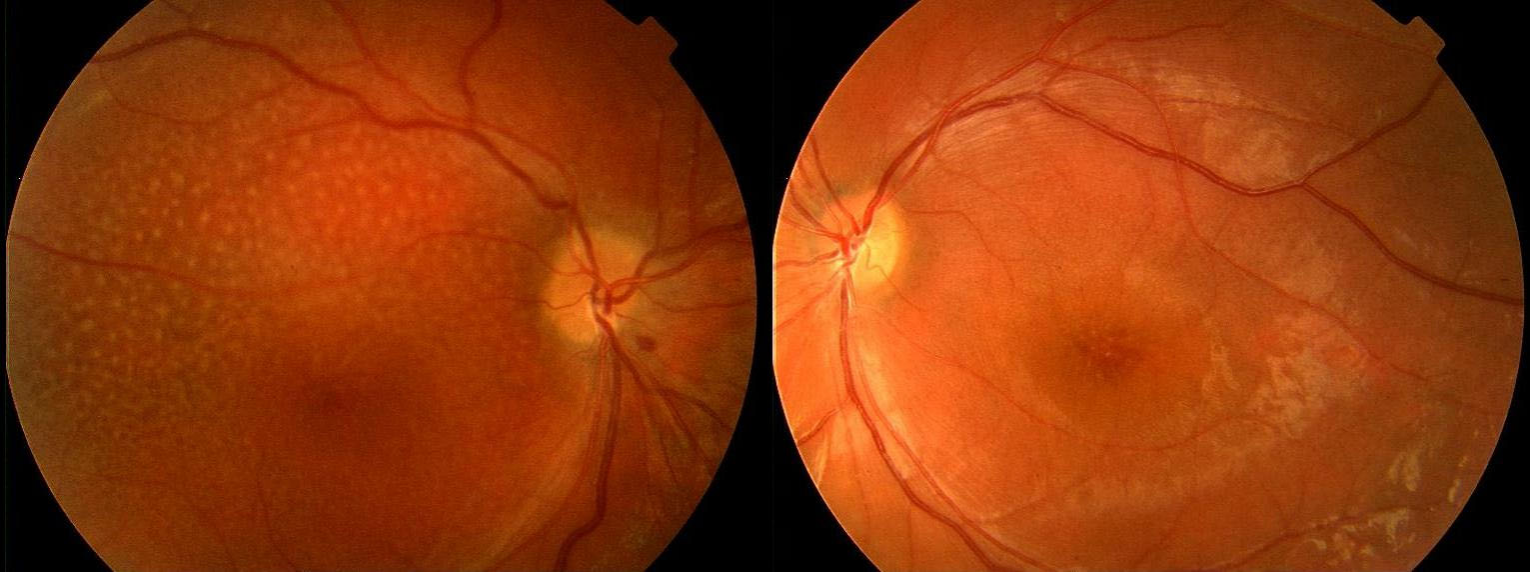
Fundus photography of patient III:3 from family 3 with c.214G>A missense mutation in the *RS1* gene. Huge foveal schisis in both eyes and pronounced white flecks, characteristic of fundus albipunctatus, were seen in the right eye and mild white dots in the left eye between the superior and inferior temporal vessels. The huge foveal schisis extended up to the vascular arcades. Pronounced bullous peripheral retinoschisis were detected in the inferotemporal part in both eyes.

Table 1, Table 2,  Table 3, Appendix 1, and the figures show the results of the clinical and molecular genetic examinations. Although impairment of BCVA was significant (p<0.05) in patients (Appendix 1) compared with the normal age-matched controls, there was no significant difference between the two patient groups. Foveal ectopy were detected in 17 eyes of 12 male patients. During a one-year follow-up only four eyes of two male patients (family 1 IV:2, family 2 III:1) showed improvement in BCVA from 0.42±0.17 to 0.27±0.13 (mean±SD). All XLRS patients had macular abnormalities seen by indirect funduscopy. Eleven of 20 patients (55%) had typical bilateral foveal schisis, 25 of 40 eyes (62.5%) had typical microcystic findings (spoke-wheel pattern; Figure 1A), and 15 eyes (37.5%) had nonspecific atrophic macular degeneration. In addition to the macular changes, peripheral retinoschisis was found only in seven eyes (17.5%) in the inferotemporal part of the retina (Figure 1B). White flecks and dots, characteristic of fundus albipunctatus, were found in two eyes of patient III:3 in family 3, and this feature was associated with the biggest foveal retinoschisis and huge bullous peripheral retinoschisis (Figure 1B and Figure 5). The distance between white flecks and dots (calculated by fundus photography) was equal to the distance between palisades connecting the splitted retinal layers (measured by OCT).

**Table 2:** Localization of retinoschisis in retinal layers

**Groups**	**Place of retinoschisis**			**Number (eyes)**	**BCVA LogMAR (mean±SD)**	**Prevalence (percent)**
	**GCL**	**INL**	**PRL**			
A	+	+	+	13	0.47±0.14	32.5
B	+	+	-	13	0.33±0.11	32.5
C	-	+	-	4	0.3±0.07	10
D	-	+	+	1	0.3	2.5

Table shows the places of retinoschisis in different retinal layers of XLRS patients detected by OCT, the number of affected eyes, the BCVA LogMAR values and the prevalence of different appearance forms. GCL represents ganglion cell layer, INL indicates inner nuclear layer and PRL refers photoreceptor layer. Group A means the presence of cysts in all 3 layers, group B means the presence of cysts in GCL and INL, group C means the presence of cysts in INL only, and group D means the presence of cysts in INL and PRL. Group A and B were the most common appearing forms. The INL was always affected in the cystic stage of the disease. The worst visual acuity was detected when cysts were located in all 3 layers (Group A).

**Table 3:** Identified RS1 mutations and their prevalence in Hungarian XLRS families

**Mutation**	**Type of mutation**	**Effect of mutation**	**Localization**	**Number of carriers**	**Number of patients**	**Number of families**	**Prevalence (percent)**
c.78+1G>C	splice site	frameshift→ truncated protein	intron 2	2	2	1	7.14
c.214G>A	missense	p.Glu72Lys	exon 4	5	5	2	17.86
c.418G>A	missense	p.Gly140Arg	exon 5	4	3	2	12.5%
c.421C>T	missense	p.Arg141Cys	exon 5	6	6+1	3	21.42+1.79
c.422G>A	missense	p.Arg141His	exon 5	5	4	1	16.07
c.574C>T	missense	p.Pro192Ser	exon 6	3	2	2	8.93
c.575C>T *	missense	p.Pro192Leu	exon 6	-	1	1	1.79
c.579dupC	insertion	frame shift→ longer protein	exon 6	3	1	1	7.14
c.625C>T	missense	p.Arg209Cys	exon 6	3	1	1	7.14
Total: 9				31	26–1=25	13	99.99+1.79

Summarized are the DNA outcome of RS1 mutations in Hungarian X-linked juvenile retinoschisis pedigrees. The following abbreviations were used: Glu is Glutamate (Glu), Arg is arginine (Arg), Gly is glycine (Gly), Cys is cysteine (Cys), His is histidine (His), Pro is proline (Pro), Ser is serine (Ser), Leu is leucine (Leu). Prevalence based on the number of persons (n=56) affected by XLRS. The illusory +1.79 value of prevalence is due to patient II:2 in family 4, who had two different mutations (c.421C>T, c.574C>T) in the *RS1* gene, which resulted in apparent prevalence greater than 100%. That is why the illusory +1 values of number of patients and the −1 value of total number of patients. The asterisk indicates a new mutation.

### Optical Coherence Tomography

Presence of cysts (any kind) was evident in 31 eyes (77.5%), 29 eyes (72.5%) had foveal cystic schisis, and 30 eyes (75%) had macular lamellar schisis. The nonspecific atrophic form in the macula (without any cystic changes) was found in 9 eyes (22.5%=type 6+7; Table 1). The most common appearance of the disease was the combined foveal cystic and macular lamellar schisis (type 3, 23 eyes, 57.5%). BCVA impaired gradually from type 1 to type 6.

The most extended foveal schisis with large, low-reflective cysts—surrounded perifoveally by many smaller ones—was located in the INL (Figure 1C). The INL was always affected in the cystic stage of the disease. Besides these, another deeper schisis was demonstrated in the photoreceptor layer (PRL), and some small cysts were seen in the GCL. Schisis was detected as follows: in 13 eyes (32.5%) in the GCL, INL, and PRL; in 13 eyes (32.5%) in the GCL and INL; in four eyes (10%) in the INL only; and in one eye (2.5%) in the INL and PRL (Table 2). The worst BCVA values were detected when cysts were located in the GCL, INL, and PRL (Group A). In case affectedness of PRL in group A, significant impairment of BCVA values were detected compared with group B (p<0.05).

The FT was significantly increased in the macula in age-group I (mean of FT±SD: 566.9±324.9, p<0.001), but there was no significant increase in age-group II (mean of FT±SD: 210.9±191.8) as compared with control subjects. TMV was significantly increased in group I (mean of FT±SD: 10±5.8, p<0.001), but there was no significant change in group II (mean of FT±SD: 6.12±1.26), compared with the controls. Significant difference (p<0.001) was detected between OCT parameters (FT, TMV) of the two patient groups. According to FT results (and similarly for TMV), the age after which the cystic form of the disease disappeared and the atrophic form occurs was 26 years (Figure 4).

### Electroretinography

**Figure 6:**
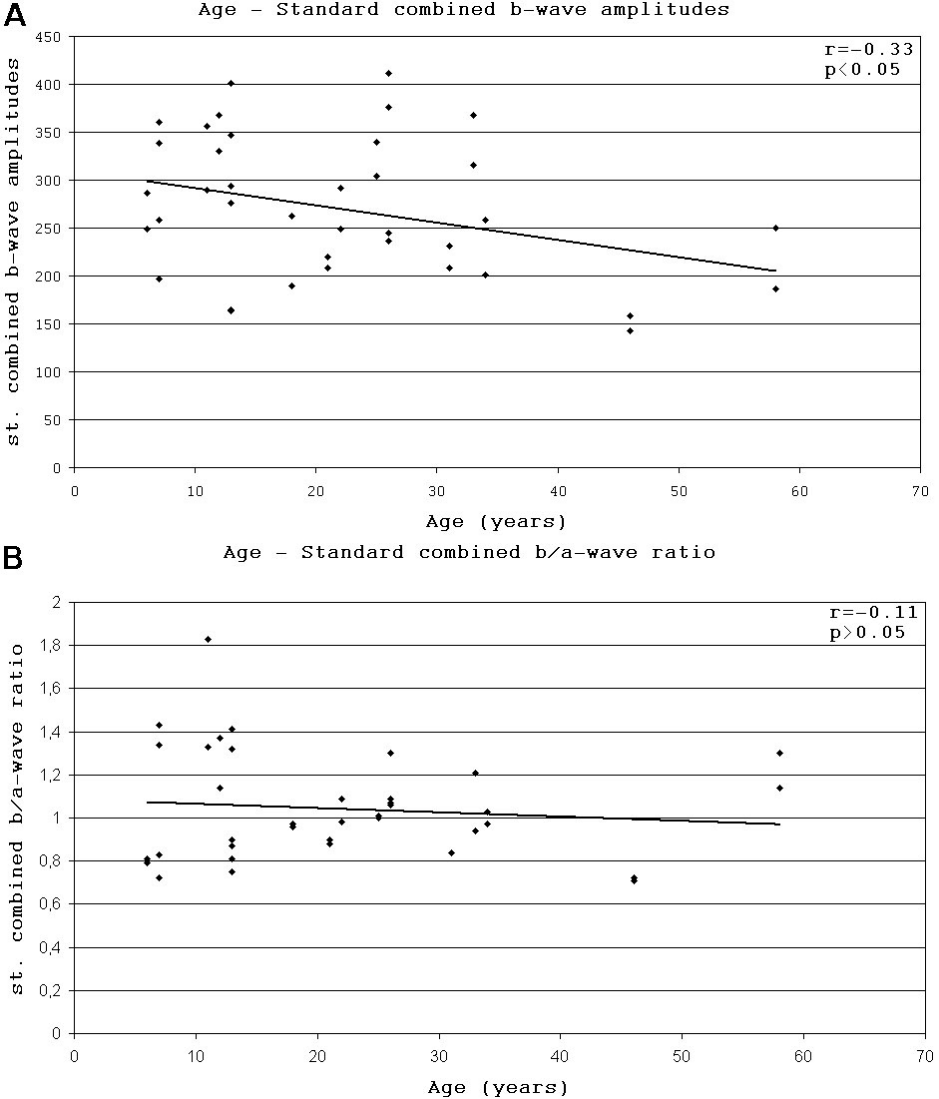
Correlation between age and standard combined b-wave amplitudes and between age and standard combined b/a-wave ratio in patients with XLRS. Black points represent eyes. **A:** Low negative correlation was detected between age and standard combined b-wave amplitudes of full-field ERG. **B:** There was no correlation detectable between age and standard combined b/a-wave ratio of full-field ERG. Negative type ERG (b/a ratio ≤1) was detected in half of the group of eyes.

All the amplitudes of scotopic (mainly standard combined b-waves) and photopic responses of full-field ERG were significantly decreased (p<0.05) in both patient groups compared with values for controls (Appendix 1). Significant difference (p<0.05) between the two age groups was found for the rod b-wave amplitudes only. The prevalence of negative type ERG was 50% (20 eyes) for all patients, but was more common (70%) in the younger group I (Figure 6B). There was no significant difference between the standard combined b-wave implicit times of both patient groups and the control values. Apart from the significantly decreased implicit times of standard combined a-waves (p<0.05), the implicit times of the other measured scotopic and photopic responses were significantly increased in both patient groups compared with the controls (p<0.05). There was no significant difference between the implicit times of younger and older patient groups.

**Figure 7:**
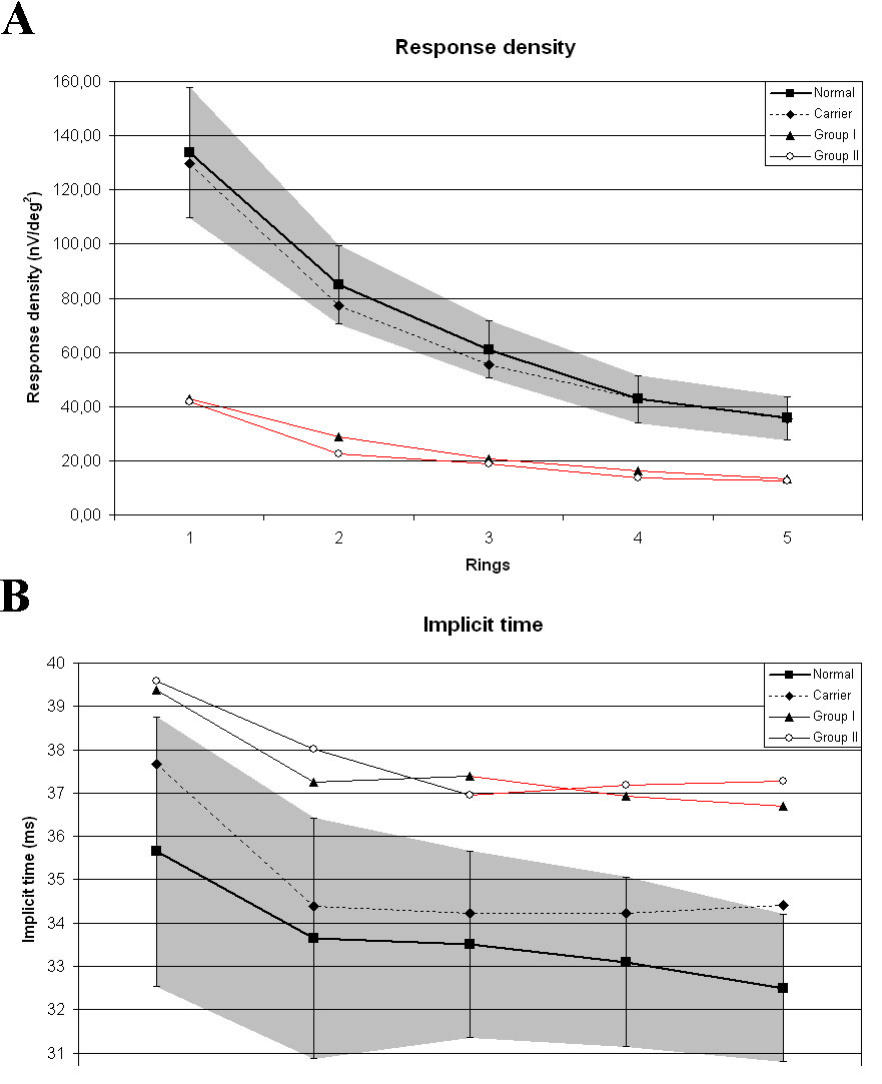
Mean values of response densities and implicit times of mfERGs for five eccentric rings in controls, carriers, and patients. Gray area represents standard deviation. Red lines highlight significant changes compared with the controls. **A:** Response densities of patients were significantly decreased (p<0.05) in all rings (especially in central rings) compared with the controls, with no significant difference found between the two patient groups. **B:** Implicit times of patients were significantly increased (p<0.05) only in the peripheral part of the examined retinal area (rings 3–5) compared with the controls, with no significant difference observed between the two patient groups. In carriers, response densities and implicit times were within the normal range.

RD of mfERG in patients was significantly reduced (p<0.001) in all rings, but mainly in the central part of the examined retinal area (corresponding to the region of foveal schisis) compared with the controls (Figure 7). There was no significant difference between the RD of younger and older patient groups. Implicit times of patients were significantly increased (p<0.05) only in the peripheral part of the examined retinal area (rings 3–5) compared with the controls. There was no significant difference between the implicit times of younger and older patient groups.

Female carriers were found to have no visual disturbances. Their BCVA (LogMAR units) were 0.0. In addition, their FT, TMV, and ERG results were within the normal age-matched ranges.

### Genetic examination

In 25 patients (mean age±SD: 26.9 years±18.5 years) and 31 female carriers (mean age±SD: 39.7 years±17.7 years) nine different mutations (eight known and one novel) were identified by molecular genetic examination (Figure 2, Table 3). Mutations, which included missense (85.7+1.79%), small frameshift insertions (7.14%), and splice site mutations (7.14%), were detected in 100% of the patients and carriers. Most of the mutations (92.85%) were localized in the conservative discoidin domain, while the RS1 domain was affected in only 7.14%. The greatest number of different mutations (four different) was found in exon 6, while exon 5 was the most frequently (49.99%) affected part of the *RS1* gene. The most commonly found mutation (23.21%) was the c.421C>T (p.Arg141Cys) missense mutation (exon 5). Molecular testing revealed a novel missense mutation (c.575C>T, p.Pro192Leu) in exon 6 of family 7. In addition, two previously reported frameshift causing mutations were found: a splice site mutation (c.78+1G>C) in intron 2 of family 2 (7.14%) and an insertion mutation (c.579dupC) in exon 6 of family 11 (7.14%). In the II:2 patient of family 4, two different mutations were found: a de novo (c.421C>T, Arg141Cys) mutation and a maternal inherited one (c.574C>T, Pro192Ser). The pathogenic effects of these mutations were confirmed by excluding their presence in 100 normal male control DNA samples.

### Correlations

**Figure 8:**
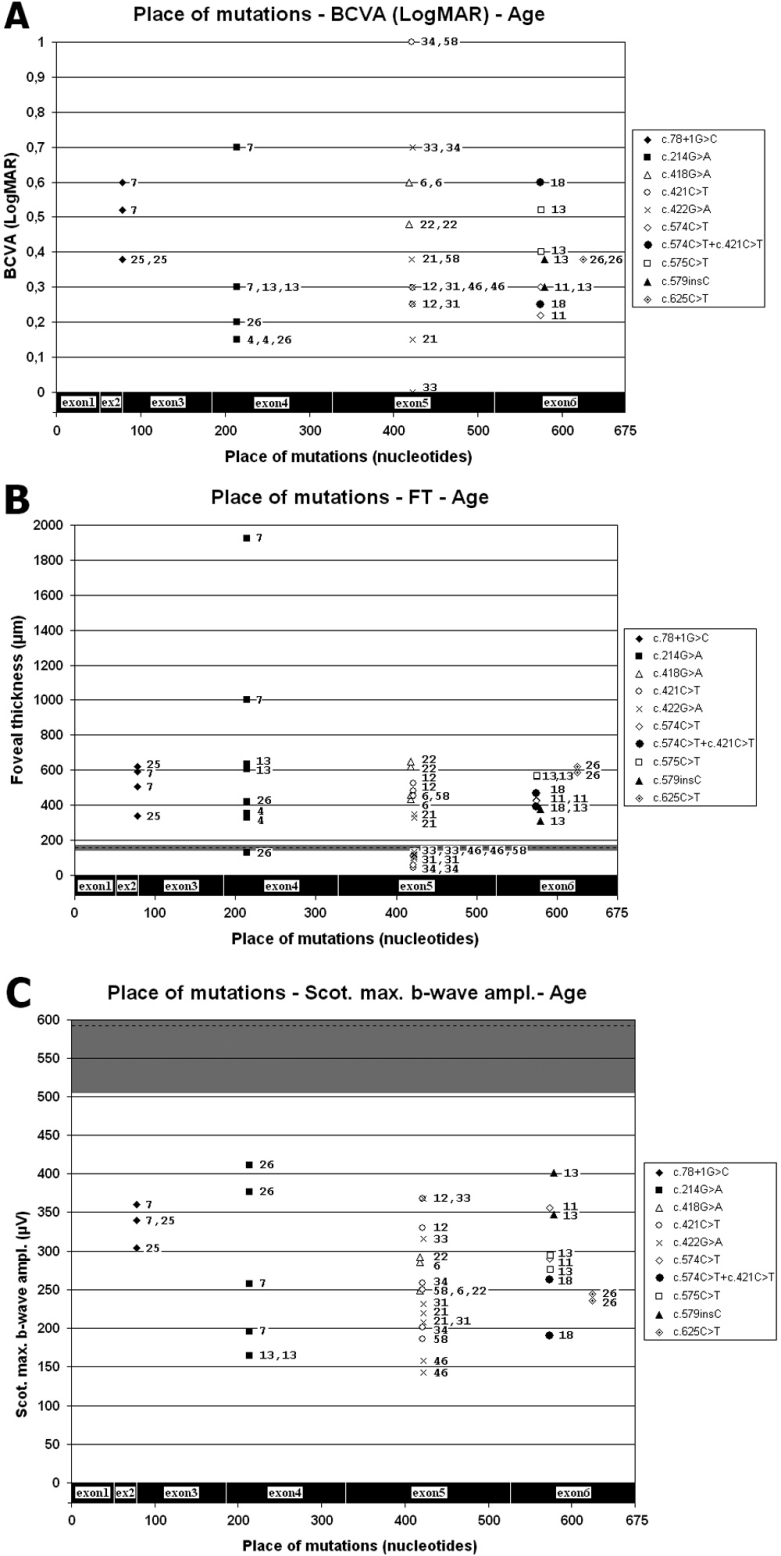
Distribution of different mutations with relevant BCVA (**A**), FT (**B**), standard combined b-wave amplitudes of full field ERG (**C**), and age. Age (yrs) is showed by numbers beside symbols characterizing the type of mutations in each eye. Mean values of controls are shown by the horizontal black broken line and the ±SD by a horizontal dark gray stripe. **A:** BCVA values belonging to a certain mutation were variable irrespective of age. The best and the worst BCVA belonged to two adjoining mutations (c.422G>A, c.421C>T). **B:** FT values change irrespectively of the type of mutation, but after 26 years of age, almost exclusively the atrophic form is detectable by OCT. **C:** The decrease of standard combined b-wave amplitudes belonging to a certain type of mutations were also different, irrespective of age.

Moderate negative correlation (r=-0.54, p<0.001) was detected between age and FT. When the stages were analyzed separately, correlation was low negative for the cystic stage (r=-0.31, p=0.14) and barely negative for the atrophic stage (r=-0.24, p=0.38) stage (Figure 4). Low negative correlation (r=-0.33, p<0.05) was detected between age and standard combined b-wave amplitudes of full-field ERG (Figure 6A). On the other hand BCVA LogMAR did not show any obvious correlation with age (r=-0.14, p=0.39), or with the type of mutation (Figure 8).

## Discussion

The BCVA of patients was significantly impaired from childhood and, contrary to the results of Pimenides et al., [21] further significant impairment was not detectable with age. Although foveal abnormalities were found in all patients using both indirect funduscopy and OCT, foveal cystic schisis was more often found (+10%) by the OCT, and appeared to mainly occur in the INL and PRL retinal layers. Aside from the presence of cysts in GCL and INL, significant impairment of BCVA was detected if photoreceptors (PRL) were affected also. The flat macular lamellar schisis was recognizable only by OCT. Presence of cysts was evident in more than 3/4 of the eyes, while the nonspecific atrophic form in the macula was found in almost 1/4 of the eyes. The high overall prevalence of the cystic form in these results is undoubtedly due to the relatively young age of our patients (mean 24.5±16.6 years). The most common phenotype of the disease was the association of foveal cysts and macular lamellar schisis (type 3). The prevalence of peripheral retinoschisis was found to be lower than generally reported in the literature [4]. White flecks and dots, characteristic of fundus albipunctatus, were associated with the biggest foveal and peripheral retinoschisis. Lesions that are like that seen in fundus albipunctatus are a known, uncommon fundus appearance in XLRS [22]. Our calculations confirmed that white lesions seemed to be identical to palisades visible by OCT between the splitted retinal layers.

Patients’ OCT images differed greatly, and a large interocular and intrafamilial variability was found. The time-course of the disease can be characterized by the OCT parameters (Figure 4). This study is the first to present the exact time of changing from the cystic to atrophic stage of the disease. Before 26 years of age, large inner nuclear central cysts (with significantly elevated FT and TMV) were characteristic (Figure 1C). After 26 years of age a nonspecific foveal and perifoveal atrophy with unchanged parafoveal retinal thickness was more common (Figure 1D). In older age the whole macula was markedly atrophic.

Regarding the change from the cystic to atrophic stage (after 26 years), a moderate negative correlation was detectable between age and FT; but within each stage there was only low and little negative correlation with age (Figure 4). Excluding the remarkable FT change about 26 years of age the aforedescribed results showed a rather limited age dependency of FT alterations. Examining the possible functional effects of the morphological changes found by OCT between the younger and older patient groups, there were no significant differences in BCVA, in amplitudes and implicit times of full-field (except the rod b-wave amplitudes) and mfERGs. The lack of correlation between OCT and BCVA was probably due to inability to fixate and the common eccentric fixation. As with other reports, we found the changing of central macular region visible by OCT was not accompanied by further visual impairment [12,23].

In two boys (family 1 IV:2, 12 years of age; family 2 III:1, 7 years of age) improvement of BCVA (three lines on the Snellen chart) was detected during a one-year follow-up, but without any notable structural changes (OCT), or functional improvement (ERG) of the retina. This was probably due to more efficient use of the ectopic parafoveal retina, and would occur only in young patients as a result of the plasticity of the developing visual system.

While previous research has demonstrated that RS1 is synthesized mainly in and secreted from the inner segment of photoreceptors [6,24–26], Takada et al. identified in healthy mouse retina that RS1 mRNA was expressed in the ganglion cells first, and later in all remarkable cells locally in the outer layers of the retina [26]. This molecular mechanism explains why retinoschisis may affect several different layers of the retina [26]. In agreement with the latest publications we found retinoschisis mainly in the INL (Müller and bipolar cells) and in the PRL, using OCT [7,9,12,27]. The dysfunctional, defective RS1 may accumulate in these layers, both intracellular and extracellular layers, leading to cysts and schisis [3,21]. In cases of nonspecific foveal atrophy, electrophysiological and molecular genetic examinations are needed to confirm the diagnosis.

Nonspecifically all the amplitudes of scotopic and photopic responses of full-field ERG were significantly decreased in both patient groups compared with the controls. The only appropriate clinical sign to confirm the diagnosis of XLRS was the pronouncely decreased standard combined b-wave amplitudes, and in advanced cases negative type full-field ERGs (in 50% of patients). Significant difference between full-field ERG responses of the two patient groups was found in case rod b-wave amplitudes only. Standard combined b-wave amplitudes of full-field ERG showed a low negative correlation with age, while BCVA LogMAR did not show any obvious correlation with age or mutation type (Figure 8).

The mainly centrally decreased P1 amplitudes of mfERG probably indicate the lack of functioning cells in the central part of the foveal schisis, while the mainly peripherally delayed implicit times may be caused by the functionally damaged but still living cells. Previous results, based on ERG examinations, found Müller cells to be primary locations of the disease [28]. Recent results demonstrate that the mfERG (FOK) P1-waves originate mainly from ON- and OFF-bipolar cells, with a smaller contribution from cone photoreceptors [28]. Thus bipolar cells seem to be affected rather than Müller cells in which, furthermore, no RS immunoreactivity was detected [24,26]. Our abnormal ERG responses also suggest that the retinal damage affects mainly the INL and the PRL layers, where the biggest schisis was found by OCT and where, normally, the RS1 can be detected in the greatest amount [12,24,25]. Flat schisis and functional impairment was detected by OCT and mfERG in a greater part of the retina than would have been expected by funduscopy.

To identify patients with uncertain diagnosis and carriers without any symptoms, molecular genetic examinations are necessary (90% certainty) [29]. On the basis of previous genetic examinations, most of the mutations are missense mutations and localized to exons 4–6 of *RS1* within the discoidin domain (dmd) [2]. Exons 1–3 tend to have mainly translation-truncating nonsense mutations [15].

This article is the first study to estimate the genetic background of patients with XLRS in Hungary. All the tested patients and carriers were found to have RS1 coding or splice site mutations. Most of the mutations were found to be localized in the conservative discoidin domain, which caused protein misfolding and finally retention of RS1 in the endoplasmic reticulum [18]. The remaining mutations were localized in the RS1 domain, which interfered with the normal oligomerization of RS1 [18]. Mutations in the leader sequence (not found in our study) prevented the translocation of the polypeptide chain across the endoplasmic reticulum membrane as part of the secretion process [18]. In each case the mutant RS1 was unable to function as an extracellular cell-adhesion protein.

Of the nine different mutations found, the c.421C>T missense mutation (exon 5) was detected most frequently. That a de novo mutation (c.421C>T) was found in patient II:2 of family 4 together with the already known disease-causing variant (c.574C>T, inherited from his mother), illustrates the remarkable new mutation rate of *RS1* and the high mutation heterogeneity of the disease. Although the aforedescribed case with simultaneous presence of two mutations and the consequent two AAs substitution led to pronounced change in the tertiary structure of the RS1 (based on the CBS CPH model), neither the clinical condition nor the examination results were affected more than in those cases with separate presence of these two mutations. It is presumed the two mutations led to intracellular degradation of the RS1 even separately [21,30]. Considering that female carriers are unaffected, it seems that the absence of a functional RS1, rather than the presence of a mutant protein, was responsible for retinoschisis in affected males [31].

We highlight the fact that a novel missense mutation (c.575C>T) was found in family 7. Although missense, insertion, and splice site mutations have also been identified, no genotype-phenotype correlation could be detected. The relatively low number of our samples, the intracellular degradation and lack of secretion of mutant RS1 (loss of protein function) could explain why phenotypes are independent of the position or type of mutation [3,21,30]. The great phenotype variability in the case of the same mutation within one family, or between two eyes of the same person, presumably reflects the influence of other factors or genes [32].

Contrary to the data of the Retinoschisis database (dmd; exon 4: 42.7%; exon 5: 16.3%; exon 6: 26.9%), the frequency of various types of mutations per exons and introns were different in these Hungarian patients (exon 4: 11.1%; exon 5: 33.3%; exon 6: 44.4%; and intron 2: 11.1%). Two mutation hot spots were found: between c.418–422 in exon 5 and between c.574–579 in exon 6. Almost 70% of the mutations were within this part of *RS1*.

Although XLRS is one of the most frequent causes of juvenile macular degeneration, it is still underdiagnosed and often misdiagnosed. Serious visual deterioration can be prevented by regular follow-up and surgical intervention in case of sudden retinal complications. Molecular genetic examinations are of particular importance to identify carriers without any symptoms and timely genetic counseling may help in family planning decisions. Although there is no current therapy for the disease, the successful gene therapy performed in mice brings hope for the future [27,31,33].

## Appendix 1. Clinical data of members

**A:** Summarized are the BCVA, OCT, full-field, and multifocal ERG values of each XLRS patients. In case of each patient the upper line represents the right eye, while the lower line represents the left eye. St. combined indicates standard combined full-field ERG response. **B:** Table shows mean values±standard deviation of BCVA logMAR, OCT, and ERG examinations in controls, carriers, and the two patient groups. Grp indicates group. To access the data, click or select the words “Appendix 1.” This will initiate the download of a compressed (pdf) archive that contains the file.
